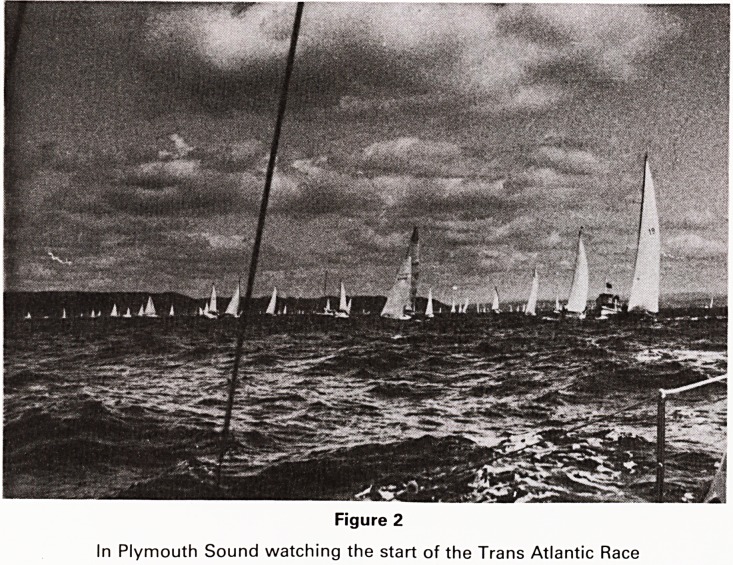# Starting to Sail

**Published:** 1986-02

**Authors:** Adrian Rozkovec

**Affiliations:** Senior Registrar in Cardiology, Bristol Royal Infirmary


### 'Starting to Sail'

**Adrian Rozkovec**
Senior Registrar in Cardiology, Bristol Royal Infirmary

My interest in sailing really started when I was houseman in Southampton. Working so near to the Solent, considered by many to be one of the best sailing areas in the country, provided an opportunity not to be missed. Although my initial trip out was in a yacht, most of my early experience was in dinghies – less expensive to buy and to repair if damaged. The first dinghy I shared was with three other equally inexperienced friends. We bought a 14 foot fibre glass family dinghy through Exchange and Mart. It seemed a bargain at the time; more of this later!

The next part of the plan was to join the local sailing club down at Lymington. For those of you who know the area, it is the unpretentious building next door to the Royal Lymington Club House. Entry to the latter requires vetting which I am told includes an interview with a real admiral. No such stiff entry criteria for us. A word with the secretary and he seconded our nominee. Being doctors may have helped although it must be said that the sea is no respecter of rank.

If you have heard of equipment failure, we had it. The second-hand outboard motor which we bought with the dinghy, exploded on the first trial. After much swearing and tinkering with the insides, it was declared inoperable, and laid to rest in a junk yard. We would regret not having one later on when we made the fundamental discovery that the sea is not a road to steer on at will. After a few trips with my ward sister, a hardy sailor, we took our first journey out into the unknown. We should have realised that the experts will make things look easy. Yes, tides were ignored and also the weather forcast. The wind died when we were somewhere in the middle of the Solent and evening was closing in with the tide taking us in stately progress away from the entrance of Lymington River. Paddling produced little headway. Fortunately, one of the few yachts out that day gave us a tow back. Most undignified. Lesson two: think before going out when there are fewer yachts on the water than expected. Dinghy sailors should not rush in where yachtsmen fear to tread.

The next lesson was called checking gear. The previous owner had sailed on a reservoir and because he was with his family, never went out in particularly windy conditions. He had filled in cracks around the base of the standing rigging (the wire stays keeping the mast up) with more fibre glass. Lesson three: when buying a boat take along someone who knows what really counts. Out in a fresh wind and sailing close-hauled, a loud cracking sound was immediately followed by collapse of the mast overboard. Fortunately, no one was hurt. Postmortem showed that the stay had pulled out taking a sizable chunk of the adjacent hull.

You might think that sailing is nothing but a frustrating experience. Not so. There can be few sailors who have not had scrapes, more so in the early stages, but as experience grows so does wisdom and respect for the sea. To make up for all those times pulling dinghies off mud, getting cold and soaked, capsizing etc., there are those glorious moments when conditions are just right and all is peace (Fig. 1). I think the feeling of calm is why such a high proportion of doctors like sailing. After the stresses of work, they want to get away from it all.

The first dinghy began to lose some of its appeal. We cold see that it was slow and did not sail close to the wind. If wind was light, others could go out when all we could do was watch. The next buy was an old second-hand Flying Dutchman. This represented the other extreme of dinghy sailing; an Olympic class boat, with a large sail area and a lot of gear. We sailed for pleasure rather than to race. In light conditions, it was a beautiful boat. Very responsive and stable. Selling it caused a period of mourning all round.

Since, I have sailed whilst in Plymouth – another beautiful coastal area but with more rugged sea conditions. Again, I bought a share in a dinghy; this time a Hornet. It became one of the talking points at the club. An unstable boat at the best of times, capsizing with ease and with a performance when coupled to its unfortunate owners to put it near the end of the racing fleet. We had much advice on altering the built-in boyancy but major surgery was not possible. Despite all this it was fun for most of the time.

I have been fortunate in knowing a few yacht owners. Yachts are those big expensive things that some people go to any length to buy even if they cannot afford it. I prefer day trips pottering along the coast, rather than long stretches with alternating four hour watches on and off. Being up at night is no longer something which I wish to do too often. I have made several trips to France and

*(continued on page 23)*

**Figure 1**

Coming in to Fowey anchorage one fine evening

### Starting to Sail *(continued from page 18)*

**Figure 2**

In Plymouth Sound watching the start of the Trans Atlantic Race sailed in Greece and Turkey. I won't bore you with any frightening experiences in yachts. Some yachtsmen delight in reciting stories of the latest dreadful conditions they have been in and with each telling the wind becomes stronger, waves larger and the lee shore approaching ever nearer. The reality should be to keep risks to a minimum. Most readers will have heard of the 'bad year' of the Fastnet Yacht Race when many people were lost. Since then, safety regulations have become much more stringent.

Many will recall the famous lines in the Wind in the Willows: 'Believe me, my young friend, there is nothing – absolutely nothing – half so much worth doing as simply messing about in boats.' Kenneth Grahame, the author, was a fanatic more married to boats than his wife. There are many examples of such indulgence both in terms of time and money. To many it is all worthwhile. One of America's cup contenders, once described the experience as like tearing up five pound notes in a shower. If you haven't sailed before, try it! (Fig. 2).

## Figures and Tables

**Figure 1 f1:**
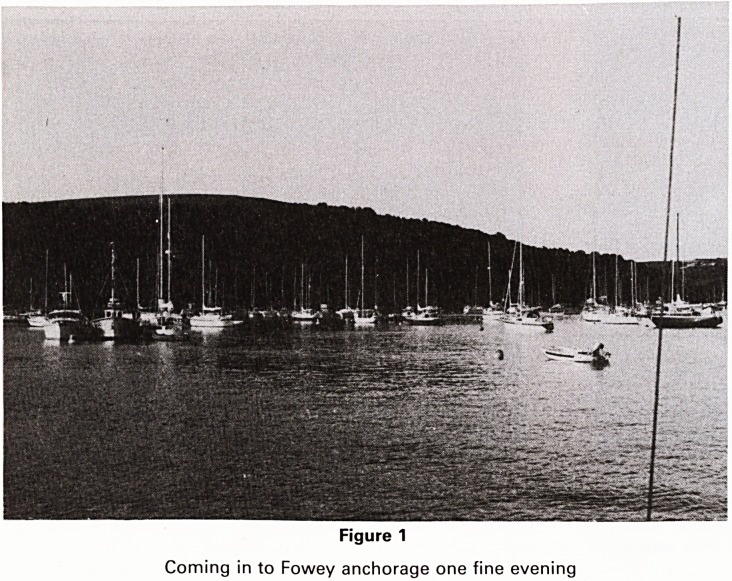


**Figure 2 f2:**